# Long non-coding RNAs in the pathogenesis of heart failure: A literature review

**DOI:** 10.3389/fcvm.2022.950284

**Published:** 2022-08-03

**Authors:** Xiaoyan Fan, Zhenwei Zhang, Liang Zheng, Wei Wei, Zetao Chen

**Affiliations:** ^1^Postdoctoral Mobile Station of Shandong University of Traditional Chinese Medicine, Shandong University of Traditional Chinese Medicine, Jinan, China; ^2^Department of Cardiovascular Disease, Affiliated Hospital of Shandong University of Traditional Chinese Medicine, Jinan, China; ^3^Department of Urinary Surgery, No.3 People's Hospital, Jinan, China; ^4^Postdoctoral Mobile Station of Wangjing Hospital, Wangjing Hospital, China Academy of Chinese Medicine Sciences, Beijing, China; ^5^Section of Integrated Chinese and Western Medicine, Shandong university of Traditional Chinese Medicine, Jinan, China; ^6^Department of Geriatrics, Affiliated Hospital of Shandong University of Traditional Chinese Medicine, Jinan, China

**Keywords:** heart failure, long non-coding RNA, pathogenesis, cardiac remodeling, cardiac hypertrophy

## Abstract

Heart failure (HF) is a common cardiovascular disorder and a major cause of mortality and morbidity in older people. The mechanisms underlying HF are still not fully understood, restricting novel therapeutic target discovery and drug development. Besides, few drugs have been shown to improve the survival of HF patients. Increasing evidence suggests that long non-coding RNAs (lncRNAs) serve as a critical regulator of cardiac physiological and pathological processes, regarded as a new target of treatment for HF. lncRNAs are versatile players in the pathogenesis of HF. They can interact with chromatin, protein, RNA, or DNA, thereby modulating chromatin accessibility, gene expressions, and signaling transduction. In this review, we summarized the current knowledge on how lncRNAs involve in HF and categorized them into four aspects based on their biological functions, namely, cardiomyocyte contractility, cardiac hypertrophy, cardiac apoptosis, and myocardial fibrosis. Along with the extensive laboratory data, RNA-based therapeutics achieved great advances in recent years. These indicate that targeting lncRNAs in the treatment of HF may provide new strategies and address the unmet clinical needs.

## Introduction

Heart failure (HF) is one of the most common cardiovascular disorders in the elderly, caused by left (or global) ventricular dysfunction, which is manifested by fatigue and dyspnea, often with signs of volume overload. Its prevalence and cost are still rising. In the guidelines for the diagnosis and treatment of HF issued by the European Society of Cardiology (ESC), HF is divided into three types according to the left ventricular ejection fraction (EF), namely, HF with reduced ejection fraction (HFrEF, EF <40%), HF with preserved ejection fraction (HFpEF, EF >50%), and the newly defined HF with mildly reduced ejection fraction (HFmrEF, 40–49%) (1). The American College of Cardiology Foundation/American Heart Association and Japanese Circulation Society/Japanese Heart Failure Society guidelines introduced a category called “HF with improved EF (HFiEF),” which is defined as patients with previous LVEF <35% and a follow-up measurement of LVEF >40% (2). Past decades have seen great achievements made in the treatment of HFrEF. However, seldom has been clinically demonstrated to improve the survival. New insights in the pathogenesis and treatment of HF are warranted (3).

Our understanding regarding the pathogenesis of HF has changed over decades. In the past, HF has been viewed as a hemodynamic disorder. Gradually, evidence unravels that activation of the renin–angiotensin–aldosterone system (RAAS) is independent of the hemodynamics and further gives birth to the neurohormonal hypothesis (4). Now, the prevailing view is that HF is a multisystemic disorder, which involves various organ pathologies, such as renal, pulmonary, and skeletal muscle diseases, as well as obesity (5). Besides, different types of HF share similarities in proposed cellular and molecular mechanisms, such as mitochondrial dysfunction, oxidative stress, cardiomyocyte and extracellular matrix-based stiffening, reduced NO bioavailability, impaired cation channel homeostasis, inflammation, and endoplasmic reticulum stress (6, 7). In the last decades, increasing evidence suggests that long non-coding RNAs (lncRNAs) serve as a critical regulator of cardiac physiological and pathological processes, regarded as a new target of treatment for heart failure. Long non-coding RNAs are transcripts exceeding 200 nucleotides in length without functional protein-coding potential.

In this study, we summarized the current advances made using the involvement of lncRNA in the pathogenesis of HF, as well as its possible clinical applications in diagnosis and treatments.

## Molecular and biological functions of lncRNAs

The Human Genome Project prevailed that the human genome compromises about 3.1 billion base pairs with only about 22,300 protein-coding genes, whereas the remaining was considered “junk DNA” (8). Consistent with this, RNA sequencing technology in recent years revealed only 2% of transcribed genomes eventually translated into proteins (9, 10). Apart from protein-coding messenger RNAs (mRNAs), transfer RNA (tRNAs), and ribosomal RNAs (rRNAs), non-coding RNAs (ncRNAs) are classified into long non-coding RNAs (lncRNAs), circular RNAs (circRNAs), small non-coding RNAs (sncRNAs), and PIWI-interacting RNAs (piRNAs) based on their structural features (11, 12).

Among these, lncRNAs are longer than ~200 nucleotides (nt), and their biogenesis is similar to mRNAs. lncRNAs are transcribed by RNA polymerase II (RNAPII) from intergenic, exonic, or distal protein-coding regions of the genome, and they often undergo 3′-polyadenylating, 5′-methyl-guanosine capping, and splicing (13). lncRNAs are versatile gene expression regulators at different levels, and their functions largely depend on interacting partners.

### LncRNAs regulate chromatin structure and accessibility

In the nucleus, lncRNAs with negative charge bind to positively charged histone tails resulting in chromatin de-compaction, which may function as a rapid switch of gene expression (14). In addition, lncRNAs directly bind to DNA forming a hybrid structure, termed triple triplexes or R-loops, which influence chromatin accessibility and DNA repair (15, 16). Recently, it was reported that NEAT1 is responsible for the assembly of paraspeckles, a form of nuclear condensates, through liquid–liquid phase separation (17).

### LncRNAs modulate transcriptional activity

lncRNA can recruit or guide chromatin modifiers, thus affecting the transcriptional activity, such as epigenetic regulatory enzymes (18). A widely known example is that HOX antisense intergenic RNA (HOTAIR) regulates chromatin dynamics and induces gene silencing *via* acting as a guide of histone methylase (PRC2) and histone demethylase (LSD1) to the correct genomic loci (19).

Besides, lncRNA can serve as a scaffold or platform at a specific genomic locus for other regulators. For instance, SPRY4-intronic transcript 1 (SPRY4-IT1) scaffolds enhancer of zeste homolog 2 (EZH2) along with LSD1 or DNA methyltransferase 1 (DNMT1) induces downstream gene silencing (20). In addition, lncRNAs compete for binding sites and prevent gene-modifying proteins from acting on the loci of interest, known as decoys.

### LncRNAs involve in translational process

In the cytoplasm, lncRNAs can alter mRNA splicing by interacting with ribosomes, competing with endogenous RNAs (CeRNA), and directly bind or sponge microRNAs (21). lncRNA, the antisense transcript of the gene ZNFX1 (ZFAS1), can interact with 40S subunit of ribosome. It is involved in ribosome production and assembly (22). Regarding CeRNA, the antisense transcript for beta-secretase-1 (BACE1-AS) competes with miR-485-5p for binding within the same region in the open reading frame of the BACE1 mRNA (23).

In addition to these functions, lncRNAs can interact with mitochondria and exosomes. lncRNAs can be encoded by mitochondrial DNA and are specifically located in mitochondria affecting mitochondrial functions and the crosstalk of mitochondria with nuclei (24, 25). A previous study found that in gene imprinting, lncRNA directly acts as a signal molecule without translation to protein, which enables a prompt response to stimulation (26). lncRNAs can also be packed into exosomes and delivered to recipient cells (27).

## Roles of lncRNAs in the pathogenesis of HF

### LncRNAs modulating cardiomyocyte contractility

A critical way for cardiomyocytes to overcome insufficient blood output is to increase their contractility. From another perspective, pathological insults impairing the cardiac contractility are considered a reason for HF.

Rhythmically, heart pumping largely relies on cellular Ca^2+^ homeostasis and excitation-contraction coupling (28). Under pathological conditions, cardiomyocytes expand contractility by elevating Ca^2+^ release from the sarcoplasmic reticulum (SR), a convoluted membrane structure serving as an iron critical storage site, into the cytoplasm (29). Cellular Ca^2+^ dynamics is controlled by two major regulators. One is the ryanodine receptors (RyRs), mediating the calcium release from the SR. The other is sarcoplasmic/endoplasmic reticulum Ca^2+^ ATPase 2a (SERCA2a), responsible for Ca^2+^ retrieval back into SR (30).

Several lines of data have revealed that lncRNAs interact with these key Ca^2+^ regulators, such as zinc finger antisense 1 (ZFAS1), myocardial infarction (MI) associated transcript (Miat), and zinc finger protein 593 antisense RNA (ZNF593-AS). Mechanistically, myocardial infarction induces the expression of ZFAS1, which is controlled by the nuclear factor of activated T-cells C2 (NFATc2). ZFAS1 binds SERCA2a and restricts its activity, thus leading to intracellular Ca^2+^ overload, abnormal Ca^2+^ transient in cardiomyocytes, and finally impaired contractile function (31). Besides, it has been identified that Miat jeopardizes cardiomyocyte calcium handling by interfering pan-RNA splicing, reducing the levels of two important calcium releasing and intaking proteins, **namely**, SERCA2a and RyR2, thereby contributing to contractility damage (32). Contrary to the mentioned lncRNAs, ZNF593-AS enhances the stability of RYR2 mRNA by recruiting HNRNPC (heterogeneous nuclear ribonucleoprotein C [C1/C2]) to the poly-U tracts of RYR2 mRNA, thereby contributing to the improvement of cardiac Ca^2+^ handling and contractile function (33). In addition, SR is physically and functionally linked with mitochondria. It controls ATP production by Ca^2+^ transferring and drives mitochondrial dynamics (34). lncRNA cardiomyocyte-enriched non-coding transcript (Caren) preserves cardiac function during pressure overload by suppressing the ataxia telangiectasia mutated (ATM)/DNA damage response (DDR) pathway, thus maintaining mitochondrial biogenesis and function (35). LncHrt modulates the deacetylase activity of sirtuin 2, which enhances the oxidative phosphorylation (OXPHOS) capacity of mitochondria, thereby preserving cardiac metabolic homeostasis and function (36).

In addition, potassium channels are key regulators in maintaining action potential prolongation and cardiac rhythm (37). Abnormal potassium channel expression results in aberrant cardiac currents and even sudden cardiac death (38). One of the major outward currents responsible for potential repolarization is the delayed rectifier potassium current (I_K_) and is dominated by Kv1.2, a subunit of the voltage-gated shaker channel family (39). An antisense RNA of Kv1.2, known as Kcna2 AS, has been shown to be elevated in cardiomyocyte hypertrophy. This lncRNA is complementary to Kcna2 mRNA, thus repressing its expression and thereby leading to malignant ventricular arrhythmias and HF (40) (Table 1).

**Table 1:** lncRNAs modulating cardiomyocyte contractility.

**lncRNA**	**Published year**	**Role in the pathogenesis of HF**	**Mechanism**	**References**
ZFAS1	2018	Promote	ZFAS1 is an endogenous SERCA2a inhibitor, impairs the contractility of cardiac muscles.	Zhang et al. (31)
Miat	2021	Promote	It interferes with Ca^2+^ transport proteins, SERCA2a, and RyR2, thereby impairing Ca^2+^ homeostasis.	Yang et al. (32)
ZNF593-AS	2021	Inhibit	It recruits HNRNPC to RYR2 which stabilizes RYR2 mRNA, improving cardiac Ca^2+^ handling and contractile function.	Fan et al. (33)
Caren	2021	Inhibit	It suppresses Hint1, which activates ATM-DDR pathway and reduces oxidative phosphorylation in cardiomyocytes.	Sato et al. (35)
lncHrt	2021	Inhibit	It interacts with sirtuin2 to preserve sirtuin2 deacetylase activity thereby ameliorating mitochondrial dysfunction.	Liu et al. (36)
Kcna2 AS	2017	Promote	It downregulates Kcna2 and attributes to arrhythmia.	Long et al. (40)

*Caren, lncRNA cardiomyocyte-enriched non-coding transcript; Hint1, histidine triad nucleotide-binding protein 1; HNRNPC, heterogeneous nuclear ribonucleoprotein C [C1/C2]); Kcna2, potassium voltage-gated channel subfamily A member 2; Miat, lncRNA myocardial infarction associated transcript; RYR2, ryanodine receptor type 2; SERCA2a, sarcoplasmic/endoplasmic reticulum Ca^2+^ ATPase 2a; ZFAS1, zinc finger antisense 1; ZNF593-AS, zinc finger protein 593 antisense RNA*.

### LncRNAs regulating cardiac hypertrophy

Cardiomyocytes are terminally differentiated cells, and therefore they cannot expand stroke work by cell division. When the contractility cannot counteract loading conditions, the cardiomyocytes synthesize excessive contractile proteins and increase in size to serve as a compensatory alternation to maintain wall stress and oxygen consumption, termed as cardiac hypertrophy (41). However, this structural change can lead to impaired subcellular organelles for efficient Ca^2+^ signaling, contraction/relaxation, and energy metabolism, and it collectively results in cardiomyocyte apoptosis, which further triggers inflammation, fibrosis, and extracellular matrix protein aggregation.

Cardiac hypertrophy has been observed in various heart diseases, such as hypertension, myocardial infarction, and valvular disease. Besides, enlarged cardiomyocytes result in ventricular hypertrophy, which increases the risk of heart failure and malignant arrhythmia. Two different types of cardiac hypertrophy have been reported. One is concentric hypertrophy, characterized by lateral growth of individual cardiomyocytes and parallel addition of sarcomeres, and the other is eccentric hypertrophy, characterized by longitudinal cellular growth and addition of sarcomeres in series (42). Mechanisms involved in pathological hypertrophy have been recognized as metabolic reprogramming, mitochondrial dysfunction, fetal gene program, and impaired Ca^2+^ homeostasis (43).

lncRNA H19 is becoming a hotspot in recent decades, due to its important role in both early postnatal development and various diseases (44, 45). H19 is a highly conserved lncRNA, transcripted from H19-insulin growth factor 2 (IGF2) locus, and rich in muscle (46). In the heart, it is mainly expressed in endothelial cells and is located in both the nucleus and the cytoplasm. Previous studies revealed its important role in various cardiovascular diseases such as ischemic myocardial injuries (47), diabetic cardiomyopathy (48), and calcific aortic valve disease (49). In heart failure, H19 is reported to be upregulated in a transverse aortic constriction model, and it inhibits cardiomyocyte hypertrophy by encoding miR-675, which targets CaMKIIδ (50). Contrary to that, another line of data finds a low expression of H19 in heart failure mice (51). Mechanistically, suppression of H19 disembarrasses the polycomb repressive complex 2 (PRC2). Then, PRC2 increases H3K27 tri-methylation of Tescalcin, followed by GSK activation and calcineurin inhibition, which finally leads to the expression and activation of NFAT, a well-known hypotrophic-promoting factor (51). Similarly, another epigenetic regulatory lncRNA, cardiac-hypertrophy-associated epigenetic regulator (Chaer), directly binds with PRC2, thereby repressing its genomic targeting ability and reducing H3K27me3 levels at the promoter regions of genes involved in cardiac hypertrophy (52). lncRNA antihypertrophic interrelated transcript (Ahit) also represses the H3K27me3 level of a critical hypertrophy-inducer, myocyte enhancer factor 2a (MEF2A). It acts as a scaffold to guide the suppressor of zeste 12 protein homolog (SUZ12) to the promotor of MEF2A, thereby mitigating hypertrophic modifications (53).

In addition to functions within nucleus, several lncRNAs have been shown to serve as the sponge of miRNA in the cytoplasm in HF. It is reported that lncRNA cardiac hypertrophy-related factor (CHRF) acts as an endogenous sponge, thus downregulating miR-489 and thereby elevating the level of myeloid differentiation primary response gene 88 (Myd88) in cardiomyocytes, and finally it contributes to cardiac hypertrophy (54). Plscr4 negatively regulates cardiac hypertrophy by sponging miR-214, which suppresses the expression of Mitofusin 2 (Mfn2) (55) (Table 2).

**Table 2:** lncRNAs regulating cardiac hypertrophy.

**lncRNA**	**Published year**	**Role in the pathogenesis of HF pathogenesis of HF**	**Mechanism**	**References**
H19	2016	Inhibit	H19 sponges miR-675 to target CaMKIIδ as a negative regulator of cardiac hypertrophy.	Liu et al. (50)
H19	2020	Promote	H19 suppresses H3K27m3 of the anti-hypertrophic Tescalcin locus which further halts NFAT expression.	Viereck et al. (51)
Chaer	2016	Promote	Chaer directly interacts with PRC2 targeting to genomic loci, thereby inhibiting H3L27m1 at the promoter regions of genes involved in cardiac hypertrophy.	Wang et al., (52)
Ahit	2020	Inhibit	Ahit serves as a scaffold to guide the SUZ12 to the promoter of MEF2A (a critical inducer of cardiac hypertrophy), leading to repressive H3K27me3 and decline in MEF2A expression.	Yu et al. (53)
CHRF	2014	Promote	CHRF sponges miR-489 and regulates Myd88 expression and hypertrophy.	Wang et al. (54)
Plscr4	2018	Inhibit	Plscr4 sponges miR-214 that targets Mfn2 thereby interfering with mitochondrial dynamics.	Lv et al. (55)

*Ahit, lncRNA antihypertrophic interrelated transcript; Chaer, cardiac-hypertrophy-associated epigenetic regulator; CHRF, lncRNA cardiac hypertrophy-related factor; MEF2A, myocyte enhancer factor 2a; Mfn2, Mitofusin 2; PRC2, polycomb repressive complex 2; SUZ12, suppressor of zeste 12 protein homolog*.

### LncRNAs involving in cardiac apoptosis

Apoptosis is an evolutionarily conserved programmed cell death that participates in embryonic development, tissue homeostasis, and pathological remodeling (56). Cardiac myocytes sustain apoptosis in response to various insults, such as hypoxia, acidosis, oxidative stress, metabolic crisis, β1-adrenergic agonists, and angiotensin II (57, 58). Cardiac myocyte apoptotic is one of the major pathological processes during heart failure. Analyses from postmortem samples reveal a small portion (<1%) of apoptotic cells in failed hearts, which is 10-to 100-fold higher than that of functional healthy control (59, 60). Apoptotic myocyte has also been shown to present in different heart failure animal models, such as ascending aortic constriction and left coronary ligation (61–63). Besides, conditionally activated procaspase-8 resulting in 0.023% of apoptotic myocardial cells generated a lethal dilated cardiomyopathy (64). Therefore, it is believed that low level, but persistent loss, of the cardiac myocyte apoptosis leads to functional limitation and finally decompensated heart failure.

lncRNA cardiac autophagy inhibitory factor (CAIF) directly binds to p53 protein and hence inhibits the activation and expression of myocardin, which suppresses autophagic apoptosis and cardiac dysfunction (65). Another protective lncRNA, myosin heavy-chain-associated RNA transcripts (MHRT), has been shown to inhibit ROS-induced apoptosis and can be used as a prognostic factor of survival in patients with heart failure (66). In diabetic cardiomyopathy, high glucose induces the expression of myocardial infarction-associated transcript (MIAT) and upregulates the death-associated protein kinase 2 (DAPK2) expression by sponging miR-22-3p, which consequently leads to cardiomyocyte apoptosis (67). It is reported that ischemia induces the elevation of sex-determining region Y-box 2 (SOX2) overlapping transcript (SOX2-OT), and hence a lncRNA sponging miR-455-3p exacerbates apoptosis rate, cell oxidative damage, and inflammatory response (68). Another ischemic-related lncRNA is H19, which has been shown to bind miR-877-3p thereby inhibiting mitochondrial apoptosis (69). Besides, lncRNA small nucleolar RNA host gene 1 (Snhg1) exerts anti-apoptotic effects on cardiomyocyte and myocardial infarction (MI) heart, by activating the PI3K/AKT/c-Myc pathway, which in turn enhances the expression of Snhg1, thus forming a positive feedback loop (70). Apart from these, cardiomyocyte mitochondrial dynamic-related lncRNA 1 (CMDL-1) has been shown to interact with dynamin-related protein 1, thereby regulating mitochondrial fission and apoptosis in cardiomyocytes (71) (Table 3).

**Table 3:** lncRNAs involving in cardiac apoptosis.

**lncRNA**	**Published year**	**Role in the pathogenesis of HF**	**Mechanism**	**References**
CAIF	2018	Inhibit	It inhibits p53-dependent expression of myocardin and autophagy induced cell death and cardiac dysfunction.	Liu et al. (65)
MHRT	2019	Inhibit	Unknown	Zhang et al. (66)
MIAT	2017	Promote	MIAT sponges miR-22-3p thus counteracting the inhibitory effect of miR-22-3p on DAPK2 and promoting cardiac apoptosis.	Zhou et al., (67)
SOX2-OT	2020	Promote	SOX2-OT sponges miR-455-3p which targets TRAF6 therefore enhances inflammation and apoptosis.	Gu et al. (68)
H19	2019	Inhibit	H19 sponges miR-877-3p thereby represses the Bcl-2 induced apoptosis.	Li et al. (69)
Snhg1	2021	Inhibit	Snhg1 elicits cardiomyocyte proliferation by sustaining PI3K/Akt signaling activation.	Li. (70)
CMDL-1	2021	Inhibit	CMDL-1 suppresses DOX-induced cardiotoxicity by regulating Drp1 phosphorylation and mitochondrial dynamics.	Aung et al. (71)

*CAIF, lncRNA cardiac autophagy inhibitory factor; CMDL-1, cardiomyocyte mitochondrial dynamic-related lncRNA 1; Drp1, dynamin-related protein 1; DAPK2, death-associated protein kinase 2; Miat, lncRNA myocardial infarction-associated transcript; MHRT, lncRNA myosin heavy-chain-associated RNA transcripts; Snhg1, lncRNA small nucleolar RNA host gene 1; SOX2-OT, sex-determining region Y-box 2 overlapping transcript; TRAF6, TNF receptor-associated factor 6*.

### LncRNAs participating in cardiac fibrosis

Consistent detrimental stimuli contribute to cardiomyocyte cell death and hypertrophic changes, which in turn prompts an inflammatory circumstance, and excessive accumulation of collagen stiffens the ventricles, which further damages the contraction and relaxation activity of the myocardium. This adverse structural remodeling is termed as cardiac fibrosis and is characterized by collagen type I deposition, as well as cardiac fibroblast activation and differentiation into myofibroblasts.

Several types of myocardial fibrosis have been reported based on etiology and pathological features: (1) reactive interstitial fibrosis, (2) replacement fibrosis, (3) infiltrative interstitial fibrosis, and (4) endomyocardial fibrosis (72, 73).

It has been well established that lncRNAs play an important role in myocardial infarction (MI)-induced cardiac fibrosis and HF, such as lncRNA-Safe (74), LncHrt (36), Wisp2 super-enhancer-associated RNA (Wisper) (75), and cardiac fibroblast-associated transcript (Cfast) (76). All of them are enriched in fibroblasts.

lncRNA-Safe elevates in both MI and TGF-β-induced cardiac fibrosis. It acts in cis by controlling the expression of neighboring gene, secreted frizzled related protein 2 (Sfrp2) (74). Wisper enhances the proliferation and survival of fibroblast and extracellular matrix synthesis, by interacting with T-cell intracellular antigen 1 (TIA1)-related protein to regulate the expression of lysyl hydroxylase 2 (75). Cfast competitively suppresses the coactosin-like 1 (COTL1) interaction with TRAP1 (transforming growth factor-β receptor-associated protein 1) and further transdifferentiation of myofibroblasts into cardiac fibroblasts (76).

However, less is known about how pressure overload-associated lncRNAs involve in the pathogenesis of cardiac fibrosis. A recent study revealed that a chromatin-associated lncRNA, lncRNA maternally expressed gene 3 (MEG3), regulates the transcriptional activity of p53 by direct interaction, which further enhances the expression of Mmp-2 and cardiac remodeling (77) (Table 4).

**Table 4:** lncRNAs participating in cardiac fibrosis.

**lncRNA**	**Published year**	**Role in the pathogenesis of HF**	**Mechanism**	**References**
lncRNA-Safe	2019	Promote	It enhances the expression of Sfrp2 and stabilizes Sfrp2 mRNA by forming a Safe-Sfrp2-HuR complex.	Hao et al. (74)
Wisp2	2017	Promote	It binds TIA1-related protein facilitating the expression of a profibrotic form of lysyl hydroxylase 2, which leads to enhanced matrix deposition and fibrosis.	Micheletti et al. (75)
Cfast	2021	Promote	It competitively inhibits the COTL1 interaction with TRAP1, which boosts TGF-β signaling by enhancing SMAD2/SMAD4 complex formation.	Zhang et al. (76)
MEG3	2017	Promote	It regulates the binding of p53 to the promotor region of Mmp-2.	Piccoli et al. (77)

*Cfast, lncRNA cardiac fibroblast-associated transcript; COTL1, coactosin-like 1; HuR, human antigen R; MEG3, lncRNA maternally expressed gene 3; Sfrp2, secreted frizzled-related protein 2; TIA1, T-cell intracellular antigen 1; TRAP1, transforming growth factor-β receptor-associated protein 1; Wisp2, Wisp2 super-enhancer-associated RNA*.

### LncRNAs mediating inflammation in HF

In addition, the inflammatory response has been shown to play a vital role in the pathogenesis of ventricular remodeling after MI. Piles of data from both laboratory and clinical aspects identify an inflammatory state, in the circulation and the heart tissue during MI with or without established HF, and have been well reviewed elsewhere (78–80). The CANTOS trial proved anti-cytokine therapy to be a promising approach toward an improved clinical outcome regarding cardiac function in patients with MI (81). However, the details of how lncRNAs mediate cardiac dysfunction remain largely unknown. One recent study revealed that SOX2-OT drives the onset of HF by sponging miR-455-3p, thereby augmenting the level of TNF receptor-associated factor 6 (TRAF6) and the activation of NF-κb signaling (68).

## Therapeutic potential of lncRNAs in heart failure

**Figure 1:**
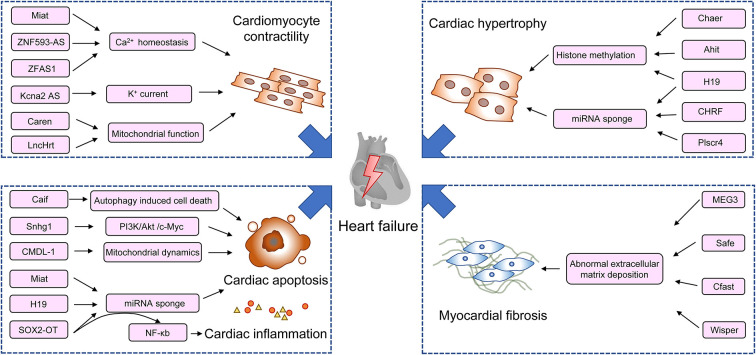
Schematic diagram of lncRNAs in the regulation of pathogenesis of heart failure. Ahit, lncRNA antihypertrophic interrelated transcript; CAIF, lncRNA cardiac autophagy inhibitory factor; Caren, lncRNA cardiomyocyte-enriched non-coding transcript; Cfast, lncRNA cardiac fibroblast-associated transcript; Chaer, cardiac-hypertrophy-associated epigenetic regulator; CHRF, lncRNA cardiac hypertrophy-related factor; CMDL-1, cardiomyocyte mitochondrial dynamic-related lncRNA 1; Drp1, dynamin-related protein 1; DAPK2, death-associated protein kinase 2; Kcna2 AS, potassium voltage-gated channel subfamily A member 2 antisense; MEF2A, myocyte enhancer factor 2a; MEG3, lncRNA maternally expressed gene 3; Miat, lncRNA myocardial infarction-associated transcript; MHRT, lncRNA myosin heavy-chain-associated RNA transcripts; Snhg1, lncRNA small nucleolar RNA host gene 1; SOX2-OT, sex-determining region Y-box 2 overlapping transcript; Wisp2, Wisp2 super-enhancer-associated RNA; ZFAS1, zinc finger antisense 1; ZNF593-AS, zinc finger protein 593 antisense RNA.

With the expansion of our understanding in lncRNAs and other non-coding RNAs, and their roles in the pathogenesis of cardiac remodeling and heart failure (Figure 1), efforts are taken to focus on their therapeutic potential. Along with the extensive preclinical data, RNA-based therapeutic development has achieved enormous advances in recent years rapidly in the field of biotherapeutics. RNA therapy is termed as an approach using RNA-based molecules to modulate specific proteins and biological pathways to treat diseases (82). It bears several advantages over traditional drugs. It can target every disease-relevant protein or gene theoretically and can be produced much faster than protein and small molecule drugs at lower costs (83). The first RNA aptamer, Pegaptanib, which is an anti-VEGF agent for the treatment of retinal diseases is licensed by the FDA in 2004 (84). After two decades of development, more antisense oligonucleotide drugs have been approved by the FDA for clinical use, such as fomivirsen, eteplirsen, nusinersen, defibrotide, inotersen, mipomersen, golodirsen, and casimersen. Another example of RNA-based therapeutic is the successful use of two mRNA vaccines, namely, tozinameran and elasomeran, which translate to a modified spike protein from COVID-19, thus facilitating a specific immune response in recipients (85). Although great achievements in RNA therapeutics have been made, the drugs to directly modulate lncRNAs' expression are still in infancy. The challenges are that several drawbacks halt the clinical translation of RNA-based therapeutics, including adverse effects, off-target activity, and aspecific delivery (12).

The future endeavor would pay on a deeper understanding of lncRNA pathology in heart failure, optimizing specificity and efficiency of the delivery system, and reducing adverse events, so as to fuel the clinical transformation of lncRNAs.

## Concluding remarks

Given the versatility of RNA properties, lncRNAs extensively participate in the cardiac physiology and pathological remodeling (Figure 1). Unraveling the details of their regulatory activities will incontestably help seek more potential therapeutic targets to supplement traditional Treatments. In addition, recent years have seen the sprouting up of innovative RNA-based technologies. The successful application of RNA-based therapeutics in the treatment of HF requires interdisciplinary collaboration, which includes chemistry, molecular biology, immunology, and pharmacology. Although challenging, with the development of the state-of-the-art technology, these laboratory efforts will pave the way for the translation of transformative therapies and to achieve accessible and personalized healthcare.

## Author contributions

WW, ZC, and LZ participated in the conception of the review. XF and ZZ drafted the initial full manuscript. WW and ZC edited the manuscript. All authors contributed to the article and approved the submitted version.

## Funding

This study was supported by the National Key Research and Development Program for Key Research Project of Modernization of Traditional Chinese Medicine (2018YFC1707402) and the Natural Science Foundation of Shandong Province (Grant Number: ZR2021MH410).

## Conflict of interest

The authors declare that the research was conducted in the absence of any commercial or financial relationships that could be construed as a potential conflict of interest.

## Publisher's note

All claims expressed in this article are solely those of the authors and do not necessarily represent those of their affiliated organizations, or those of the publisher, the editors and the reviewers. Any product that may be evaluated in this article, or claim that may be made by its manufacturer, is not guaranteed or endorsed by the publisher.
